# Regulatory requirements for the registration of generic medicines and format of drug dossiers: procedures in Sri Lanka in comparison with selected regulatory authorities

**DOI:** 10.1186/s40545-018-0141-2

**Published:** 2018-06-21

**Authors:** D. Thambavita, P. Galappatthy, R. L. Jayakody

**Affiliations:** Department of Pharmacology and Pharmacy, Faculty of Medicine, University of Colombo, Kynsey Road, Colombo, 08 Sri Lanka

**Keywords:** Dossier, Generic drugs, Bioequivalence, Biowaiver, DRA process

## Abstract

**Background:**

The regulatory requirements for approval of generic medicines and the format of compiling drug dossiers vary among regulatory authorities. The variation is particularly wide between High-income countries (HIC) and lower and middle-income countries (LMIC) with different regulatory frameworks. In this study, document requirements for approval of generic products, approval timelines, and consideration of bioequivalence and/or biowaiver data by Regulatory Authorities (RAs) of 10 selected jurisdictions was studied.

**Methods:**

The guidelines and procedures from 5 purposively chosen RA of HIC and4 regional RAs relevant for Sri Lanka were compared with the Sri Lankan National Medicines Regulatory Authority (NMRA). Information available in the official websites of the selected RAs, published journal articles and via personal communication was collected in2016. Drug approval timelines achieved in Sri Lanka was obtained from data available from another study.

**Results:**

Common technical dossier (CTD) format of the International Council on Harmonization (ICH) for registration of pharmaceuticals (ICH:CTD) or the Association of South East Asian Nations (ASEAN) CTD format (ACTD) was used by all RAs studied except Sri Lanka which use its own dossier format. Nine out of ten RAs studied request BE data or justification for not submitting BE data for generic medicines. Sri Lanka requested BE studies only for antimicrobials, antiepileptic drugs and narrow therapeutic index drugs. Biowaivers are allowed for Biopharmaceutics Classification System (BCS)-based Class 1drugs in Singapore and India. USA, EMA, Canada and South Korea allowed biowaiver for BCS Class1and Class 3drugs but Sri Lanka does not accept BW at present. Nine NMRAs out of the ten studied reported legislated timelines for approval of generic pharmaceuticals except Sri Lanka.

**Conclusions:**

Streamlining the drug regulatory systems in LMIC such as Sri Lanka with that of HIC would facilitate an effective drug regulatory system based on reliance on decisions made by stringent regulatory authorities. Findings of this study encourage Sri Lanka to adopt a CTD format for regulatory submission of drug dossiers. Expanding the BE requirement drug list and accepting BCS-based biowaivers for BSC class 1 and 3 drugs during registration of generic drugs when it is scientifically justified is also recommended for Sri Lanka.

## Background

The primary goal of a national medicine Regulatory Authority (RA) of a country is to approve efficacious, safe and quality drugs to the people of the country. The RAs meet two types of drugs, viz., new drugs (NDs) and generic or multisource medicines. With increasing use of generic medicines, a considerable amount of time of a RA would be spent on evaluating and approving generic medicines. Evaluation of the quality of the generic products becomes crucial for RAs of the lower and middle-income countries (LMIC). The situation becomes more complicated with biologics and bio-similar products, as they are not identical to innovator products. During the regulatory review process, the interchangeability of the generic product with the originator product was evaluated. Most RAs use bioequivalence (BE) data when evaluating their interchangeability [1–4]. BE is determined by administering the generic and a comparator medicine to a group of healthy volunteers and measurement of serial blood concentrations over a 24-h period. Most RAs recommend either the innovator product or an already approved brand-named product to use as the comparator product. As BE studies involve human volunteers and measurement of very low concentrations of the drug in blood they are very expensive to carry out. Moreover, the RAs will need to have experienced evaluators to evaluate BE data and to check their authenticity. To overcome some of these difficulties, the concept of biowaiver (BW) was introduced based on the Biopharmaceutics Classification System (BCS) of Active Pharmaceutical Ingredients (APIs) which can be applied for Immediate Release (IR) solid oral generic drug products. In BW studies, comparative dissolution testing is carried out between a comparator and generic product in 3 different pH media representing gastric, duodenal and intestinal pH. These in vitro BW studies are much cheaper to carry out when compared to the in vivo BE studies particularly for LMIC [5]. Therefore, most countries including the High Income Countries (HIC) are now resorting to accept BW data in place of BE data for selected drugs [4, 6–8]. Granting BW is based on the solubility and permeability of the API, and in vitro dissolution of the finished product. However, one of the challenges faced by RAs of LMIC is lack of properly trained, experienced technical staff to evaluate the BE/BW data, interpret and advice on the data. Thus, some RAs in LMIC have regulatory systems that rely on the review process already done by ICH member countries or ICH observer countries. This process would be easier if a common dossier format is used during regulatory submission. Another factor that is important to the industry is to know how quickly they can get the registration of their products. Thus, most RAs have published drug approval timelines in order to establish an efficient and transparent drug regulatory process in their countries. They revise these timelines based on past performance [9].

Sri Lanka is a LMIC in South Asian region and vast majority of the medicines used in the country are generic medicines. The medicines regulation in Sri Lanka underwent a major revision recently with the implementation of a new National Medicines Regulatory Authority Act of 2015 passed in parliament and the establishment of a new National Medicines Regulatory Authority (NMRA) in 2015 [10]. The NMRA replaced the Cosmetics Devices and Drugs Authority (CDDA) and the CDDA Act of 1982. Although the establishment of the NMRA is considered a significant improvement in medicines regulation in Sri Lanka, we believe that the medicines regulatory system in Sri Lanka could be further improved. In this background, this study was planned to study the generic medicines approval process in Sri Lanka, compared to the regulatory process used in purposively selected 9 other countries. The generic medicine approval process was compared on the use of a common technical dossier (CTD) and the acceptance of BE and BW data. In addition, the legislated timelines for registration, the target time lines and achieved time taken for both generic and new drug product approvals was studied across the selected RAs. We also planned to make recommendations to the Sri Lankan NMRA to improve its generic drug approval process based on our findings.

## Methods

The purposively selected RAs for the study included those generally considered as ‘stringent’ regulatory authorities (SRAs) and the regional authorities that are relevant to Sri Lanka, from which it was possible to obtain data required from the official websites. The definition of ‘stringent’ used by the Global Fund was used in this study [11]. Accordingly, SRA means a regulatory authority which is (a) a member of the ICH or (b) an ICH Observer, being the European Free Trade Association (EFTA) as represented by Swiss Medic, Health Canada and World Health Organization (WHO) or (c) a regulatory authority associated with an ICH member through a legally binding mutual recognition agreement including Australia, Norway, and Iceland. Using this definition of SRAs, as all ICH member countries have similar or comparable generic medicine approval systems, three jurisdictions from ICH-member countries, one from ICH-observers and one from ICH-associate countries were selected as the five SRAs for the study. The international SRAs included were three ICH- members i.e. European Medicines Agency (EMA), United States Food and Drug Administration (US FDA) and Pharmaceuticals and Medical Devices Agency (PMDA) of Japan, one ICH-observer i.e. Health Canada (HC), and one ICH-associate i.e. the Therapeutics Goods Administration (TGA) of Australia. The four regional agencies selected were Central Drugs Standard Control Organization (CDSCO) of India, National Pharmaceutical Control Bureau (NPCB) of Malaysia, Health Sciences Authority (HSA) of Singapore and Food and Drug Administration of South Korea (KFDA). Requirements of these RAs were compared with the processes adopted by the NMRA of Sri Lanka.

Data were collected from July–December 2016 for the comparison study from the selected RAs. Data for the achieved drug approval time in Sri Lanka was obtained from data available from another study conducted by the authors from January to May 2014 [12]. The generic medicine approval process comparison was conducted through the information available on the official websites of the selected NMRAs and published articles in books and journals. Journal articles were searched using Google Scholar and PubMed. Recommended drug dossier format for regulatory submission of generic medicines, use of BE and BW data were compared in the websites of selected RAs and data available from published literature. Information about the NMRA, Sri Lanka was obtained through documents available with the officials of the NMRA. Legislated drug approval times and targeted drug approval times of these jurisdictions were obtained for both generic medicines and New Drug Application (NDA). Data on these drug approval times were obtained from the official websites and achieved drug approval times were obtained from published literature. As there were no mandated or targeted timelines available for Sri Lanka, achieved median drug approval time for Sri Lanka was calculated using unpublished primary data collected for another study conducted by the authors [13]. In that study on quality of registered amoxicillin products’, data on 8 imported generic amoxicillin dossiers were audited based on ‘clock- stop’ method excluding client’s time [14]. The approval time considered was from the date of submission of the dossier until the date of approval of the medicinal product. The Median time for generic medicine registration was calculated using the time taken for registering these generic dossiers of imported products of amoxicillin. For other RAs, the drug approval times reported in the official websites and literature were obtained and reported. Guidelines and regulations pertaining to drug approval were also obtained from the official websites of the selected jurisdictions.

## Results

### Definition of generic drugs used by RAs

Definition and subsequent explanations provided for ‘generic medicine’ by the selected RAs in their guidelines contained common criteria [7, 8, 10, 14–19] of active drug substance being qualitatively and quantitatively same with an already approved product, having the same or comparable dosage form, having the same route of administration, and demonstrating bioequivalence with a reference product.However, there were differences in consideration of salts or esters as the same active (or drug) substance, in the reference product. With regards to active pharmaceutical ingredient, USA, EU, Australia and Singapore accept different salts as the same active (or drug) substance, if it is the same active moiety in the reference product. However, Japan does not consider different salts as the same active substance.

### Generic drug dossier format

Nine out of the ten RAs studied were using one of two common technical dossier (CTD) formats. Australia [20], Canada [21, 22], EU [23], India [24, 25], Japan [26], US [27] and South Korea [28] were using the ICH:CTD format. Association of Southeast Asian Nations (ASEAN) CTD format (ACTD) was used by Malaysia [29]. Singapore accepted dossiers in either formats [30]. Sri Lanka was using a format which it had developed based on the WHO recommendations for drug registration but not claimed to be either ICH:CTD or ACTD [31]. This drug dossier format used in Sri Lanka consisted of 79 criteria out of 126 recommended by the WHO [32]. ICH:CTD and ACTD formats are similar in many aspects as the ACTD format has been derived from ICH:CTD format. In the ICH: CTD format, information should be organized into 5 modules. The ACTD format consists of four parts which corresponds to modules 1, 3, 4 and 5 of the ICH: CTD format. ACTD format does not have a part corresponding to Module 2 of the ICH: CTD, which contains summaries of Modules 4 and 5. Data on animal and clinical studies and bioavailability studies are included in Modules 4 and 5 of ICH: CTD and in Parts III and IV of the ACTD format for innovator medicines. These are replaced by the bioequivalence data in generic drug dossiers. Of the 10 NMRAs studied, except Sri Lanka all other nine DRAs accept drug dossiers through either electronic version of the CTD format (eCTD), or data via e-Drug Service in South Korea (ezfiling) or electronic filing of the data submitted through hard copies in India.

### Requirement of bioequivalence data

All the RAs except Sri Lanka used their own or adopted guidelines in establishing interchangeability of generic drugs but in Sri Lanka, there is no written BE guideline. Australia, EU, India and Canada required BE data at the time of registration for all solid oral dosage forms of generic drugs (Table 1). Therefore, for a drug to be qualified as a ‘generic’, it needed to demonstrate either in vivo or in vitro BE (for drugs for which BCS-based BW is accepted) with the originator product. Singapore requires BE for all prescription only medicines (PoMs) and Japan requires in vivo BE for first approval of all generic drugs. Australia requests BE or justification for not submitting BE for all PoMs. For over the counter (OTC) medicines they have given a list of medicines not requiring BE. Since 2014 Sri Lanka requires BE data for antimicrobials, antiepileptic drugs and narrow therapeutic index drugs as per a circular issued by the NMRA. However, NMRA, has not given a definition that it uses for narrow therapeutic index drugs but the drugs that are generally considered to have a narrow therapeutic index as given in standard text books are considered under this category during their regulatory decisions on “case by case basis”.

**Table 1:** Bioequivalence data requirements and biowaiver data acceptance by the selected RAs

Jurisdiction	Bioequivalence data requirement	Biowaiver data acceptance
Australia	POM and for an identified list of OTC medicines.	BCS Class1drugs
Canada	Required for all solid oral generic drugs	BCS Class1 and Class 3 drugs [7]
EU	solid oral generic drugs	BCS Class1and Class 3 drugs [46]
India	solid oral generic drugs	BCS Class 1 drugs and Schedule Y drugs^a^ and drug already approved for new claims, new indication, new dosage form/ new route of administration, modified release dosage form [25]
Japan	solid oral generic drugs. (In vivo BE is required for first approval of all generics).	Does not accept BCS-based biowaiver for first approval of generic drugs. Accepts dissolution studies^b^ for minor changes in the formulation and the dose strength
Malaysia	solid oral generic drugs	BCS Class 1drugs. A list for which biowaiver can be considered is provided [16, 29]
Singapore	POM and for over the counter medicines not included in the list of medicines exempted from BE	BCS Class 1drugs and for additional strengths of the drug if doses are proportional [8]
South Korea	solid oral generic drugs	BCS Class 1 and Class 3drugs
Sri Lanka	antiepileptic, narrow therapeutic index drugs, sustained release products and antimicrobials (from 2014)	Does not accept BW data
USA	solid oral generic drugs	^c^BCSClass1and3drugs [6, 47, 48]

^a^ New drugs which require human clinical pharmacology data for registration under the rules for Schedule Y of Drugs and Cosmetics rules of Central Drugs Standard Control Organization (CDSCO), India

^b^ Multimedia dissolution tests

^c^ For first time registration

### Requirement of biowaiver data

USA, Canada, EMA and South Korea accept BCS-based biowaiver for both BCS Class1and Class 3drugs. Australia, India, Malaysia, and Singapore accept BCS-based BW for BCS Class 1drugs (Table 1). Japan does not accept BCS-based BW when registering generic drugs for the first time. It accepts dissolution test results for minor changes in the formulation and the dose strength, which is allowed for all drugs. Sri Lanka does not have a policy on accepting BW.

### Time taken for generic drug registration

Generic drug approval timelines identified and reported from the selected RAs are given in Table 2. Accordingly, 9 out of the 10 RAs studied had mandated drug approval timelines. Achieved times from studies were available only for Sri Lanka and Japan. According to the Melchior [33], in the USA, the target given for standard drug approval for 2010 was 10 months. The performance goal for both USA and Canada for the fiscal year 2010 was to act on 90% of the submission within 10 months [33, 34]. Australia has legislated timelines for drug approval within 40 working days (WD) commitment for informing acceptance or rejection for processing. The, legislated TGA commitment was 255 WD and it was being streamlined to have no more than 300 CD (10 months), including the sponsor time [33]. The 12 months drug approval time in Japan includes 1.5 months applicant response time and 2 months for Good Manufacturing Practices (GMP) inspection time [35]. In practice, the reported regulatory review time was 9 months excluding applicant time in the year 2013 in Japan [33]. Singapore and Malaysia conduct abridged drug dossier evaluation. In Singapore, the approval time for verification dossiers, which are already evaluated and approved by EMA, US FDA, Health Canada, TGA, and UK MHRA is only 60 WD for new drug applications or 120 WD for generic drug applications. According to the reported data, EU has a centralized drug evaluation process with EMA which has legislated timeline of 210 WD. (this is subjected to certain specific National Authority timelines within EU) [33] Malaysia takes 12 months for evaluation of hard copy submissions and 6 months for online submissions [33]. Sri Lanka did not have published legislated or targeted timelines for drug approval. Thus, the study conducted based on an audit of 8 generic dossiers showed median time of 90 WD with the range of 7 to 379 WD for Sri Lanka.

**Table 2:** Drug approval timelines of the selected NMRAs

Jurisdiction	Legislated drug approval time		Targeted drug approval time		Achieved drug approval time	Reference
	NDA ^g^ or NCE ^h^	ANDA ^i^ or generic medicines	NDA or NCE ^h^	ANDA ^i^ or generic medicines	ANDA ^i^ or generic medicines	
USA	6 months under priority review 10 months under standard review	180 WD	To act on 90% of the submissions within given time	To act on 90% of the submissions within given time		[33]
Australia	255 WD ^c d^	175 WD ^c d^				[49]
Canada	300 CD ^a^	180 CD ^a^	To act on 90% of the submissions within given time	To act on 90% of the submissions within given time		[50]
EU	210 WD ^c^ 150 WD ^c^ (accelerated assessment)					[51]
Japan	15.3 months	12 months ^d^			9 months in the year 2013	[52]
India	180 days	120 days				[53]
Malaysia	245 WD ^c d^ (Full evaluation)	210 WD ^c d^ (Full evaluation) 116 WD ^c d^ (Abridge Single active ingredient) 136 WD ^c d^(Abridge two or more active ingredients)				[2]
Singapore	180 WD ^c^ (abridged evaluation) 270 WD ^c^ (full evaluation) 60 WD ^c^ (for verification dossiers)	240 WD ^c^ (abridge evaluation) 120 WD ^c^ (for verification dossiers)				[30]
Republic of Korea	30 days for INDs ^e^ 90 days when DMF ^f^ review is not included 120 days when DMF ^f^ review is included					[28]
Sri Lanka					90 days (generic medicine)	

^a^ CD Calendar dates

^b^ The performance goal for the year 2010 was to act 90% of this given timeline

^c^ Working days

^d^ including client time

^e^ Investigational New Drug

^f^ DMF: Drug Master File

^g^ NDA- New Drug Application

^h^ NCE- New Chemical Entity

^i^ ANDA – Abbreviated New Drug Application

## Discussion

All selected RAs referred to pharmaceutical equivalents to describe term ‘generic medicine’ in their guidelines. Thus, pharmaceutical equivalents contain identical active drug ingredients which may also can be presented either in same salts or ester forms of the same therapeutic moiety. This definition was similar across the RAs studies except in Japan.

A CTD format was used by all RAs studied except Sri Lanka, when submitting generic drug dossiers for registration. Even though ICH countries initially worked on evaluation and registration of products containing NDAs and new biotechnological products in ICH countries, their guidelines now address quality of generic products [36]. Thus, later versions of ICH guidelines have influenced the generic drug manufacturers in other countries. Notwithstanding the difficulties of implementing highly technical scientific requirements which require considerable cost, there are other countries that have adopted the ICH guidelines [37]. This adoption has given wider acceptability to the ICH standards [37]. According to our study results, three out of four regional RAs selected for the study, i.e. India, South Korea and Singapore, use the ICH: CTD format. Two out of the ten RAs studied used the ACTD format for regulatory submission. ASEAN works on developing reference substances, guidelines and trade agreements with regards to pharmaceuticals in the ASEAN region [38, 39]. The ACTD format aims at establishing a common technical document to harmonize pharmaceutical product dossiers with the ICH [39]. As most of the LMIC in Asia get at least some of their drug products already registered in an ICH jurisdiction, a detailed review of efficacy and safety data is often not required. Further, a country like Sri Lanka can benefit from converting to a CTD format as this facilitates approval based on reliance of judgment made by a SRA.

The local dossier format currently used by the NMRA, Sri Lanka had been developed based on WHO technical guidance at its start in the 1970s [40]. No official updated guidance has been issued since then on the dossier format or other requirements such as BE. Hence the details of BE requirements have so far not been incorporated into it. However, the new NMRA Act of 2015 states that regulations may be made in respect of BE and BW data related to generic medicines submitted for evaluation [10]. Our findings encourage Sri Lanka to have regulations to be made on a CTD format for regulatory submission of drug dossiers. Results also indicate leading well-resourced agencies internationally and regionally facilitating electronic submission of drug dossiers.

Nine out of the ten RAs studied except Sri Lanka had guidelines on BE data requirements. Accordingly, BE data is a mandatory requirement particularly for all prescription only medicines during solid oral generic medicine registration. In vivo BE studies of generic medicines provide greater assurance of BA/BE and consistent therapeutic outcomes. However, BE requirement affects the product cost and timely availability of generic products. The BCS-based biowaiver for solid oral generic products exempts generic drugs belonging to certain BCS classes, from expensive and cumbersome in vivo BE studies. According to Davit et al., presently USA and EU waive in vivo BE for a different strength of BCSClass1 and Class 3drugs which are already approved by those jurisdictions [41]. It further highlights better harmonized BCS-based BW guidance among US FDA, EU and the WHO [41]. EU also considers BW for modified release dosage forms for which acceptable in vitro*-*in vivo correlation (*IVIVC*) is established [42]. Four RAs studied allow BW for both BCS Class 1 and Class 3 drugs, while five RAs studied allow BW only for BCS Class 1drugs. All RAs in their guidelines documents on application of BW have clearly stated that BW should not be allowed for narrow therapeutic range drugs. The study revealed that the NMRA of Sri Lanka presently does not have any guidelines on application and acceptance of BW. Although the definition of ‘generic drug’ indicates already proven bioequivalence with a reference product, in Sri Lanka according to the circulars issued only antibiotics, antiepileptic drugs and narrow therapeutic index drugs need to show evidence of bioequivalence. Careful control of the interchangeability of generic medicines is mandatory to have safe generic substitution. As Sri Lanka depends heavily on generic medicines, regulatory decisions are needed for the application of BCS-based biowaiver in establishing interchangeability and waiver of in vivo BE studies for generic medicines. Thus, if Sri Lanka can explicitly apply BCS-based BW it will particularly support the local pharmaceutical manufacturing industry in registering their generic pharmaceuticals by allowing demonstration of interchangeability using low cost BW studies. Furthermore, having guidelines for the industry will facilitate more transparent and effective regulatory system.

Generally, the approval process of a generic drug is considerably simpler and less time consuming compared to a NDA. The drug approval process could be a standard review or expedited review (may be differently named by different RAs). According to Kashyap et al., in the EU, generic drugs are mainly approved through a mutual recognition procedure which has a timeline of 390 days [43]. According to the results of our study nine out of the ten jurisdictions had mandated drug approval timelines. A comparison study on the regulatory review process across five regulatory agencies in the USA, Europe, Canada, Switzerland, and Australia reported overall approval time range across these jurisdictions, which ranged from 368 to 595 days including client times [44]. However, this comparison was conducted in 2007 and now the actual timelines of these jurisdictions may be different. NMRA Sri Lanka did not provide approval timelines. The median drug approval time reported as 3 months from Sri Lanka, was based on the results of an audit conducted on 8 generic drug dossiers. The NMRA of Sri Lanka has registered a considerable number of pharmaceutical products in the recent past [45]. The, the shorter drug approval times indicate expedited review coupled with non-requirement of BE data for most drugs during the generic drug approval in Sri Lanka. Having legislated or targeted drug approval timelines show the transparency in the regulatory process and it is a measure of regulatory efficiency. On the other hand, imposition of hard, legislated time targets could lead to decision errors due to hurried evaluation with adverse consequences. Therefore, instead of unrealistic timelines, it would be better to have performance based time targets for drug approval which would pave the way towards an efficient and timely drug approval process.

Although initial harmonization work was more with NDAs [9] more recent literature by Davit et al. discussed good convergence of US FDA, EU and WHO on BCS biowaiver criteria as applied to generic medicines [41]. Harmonization of the procedures and documentation improved the reliance on stringent regulatory systems. Having an effective drug regulatory system will pave the way towards mutual recognition of regulatory activities among RAs, which would ultimately help to ease the regulatory burden of jurisdictions with limited resource. Although this study highlighted the areas for improvement in generic drug registration process in Sri Lanka, the registration process of biologics, biosimilars, and NCE were not studied. Regulatory timeline was also obtained only for a limited number of generic products registered Sri Lanka. Therefore, further studies are needed to identify registration process for the other categories of medicines and the actual drug approval timelines for a larger number of dossiers submitted for registration in Sri Lanka.

## Conclusion

This study evaluated the drug registration process and requirements for registration of generic medicines in Sri Lanka, a LMIC in South Asia, to provide recommendations to NMRA. Adopting a CTD format for regulatory submission and enabling electronic submission would facilitate regulatory review process by speedy review, easy communication with the applicant, reducing regulatory burden and also improving industry compliance. The list of medicines that require BE data needs expansion in Sri Lanka to ensure the quality of generic drugs registered. NMRA should also apply the concept of BCS-based BW for BCS Class 1 and Class 3 drugs when it is scientifically justified. Therefore, we recommend the NMRA Sri Lanka to adopt a CTD format for regulatory submission of drug dossiers, facilitate electronic submission, expand the list of drugs requiring BE and accept BCS based BW for BCS class 1 and 3 drugs when scientifically justified.

## Abbreviations

ACTD: Association of Southeast Asian Nations’ common technical dossier
API: Active pharmaceutical ingredient
ASEAN: Association of South East Asian Nations
BCS: Biopharmaceutics Classification System
BE: Bioequivalence
BW: Biowaiver
CD: Calendar days
CDDA: Cosmetics Devices and Drugs Authority
CDSCO: Central Drugs Standard Control Organization
CTD: Common technical dossier
eCTD: Electronic common technical dossier format
e-filing: Electronic filling
EMA: European Medicines Agency
EU: European Union
GMP: Good Manufacturing Practices
HC: Health Canada
HSA: Health Sciences Authority
ICH:CTD: International Council on Harmonisation of Technical Requirements for Registration of Pharmaceuticals for Human Use Common Technical Dossier format
IR: Immediate release
IVIVC: In vitro-in vivo correlation
KFDA: Korean Food and Drug Administration
NCE: New chemical entity
NDA: New Drug Application
NMRA: National Medicines Regulatory Authority
NMRAs: National Medicine Regulatory Authorities
NPCB: National Pharmaceutical Control Bureau
OTC: Over the counter
PMDA: Pharmaceuticals and Medical Devices Agency
POM: Prescription only medicines
RA: Regulatory Authority
SRAs: Stringent regulatory authorities
TGA: Therapeutics Goods Administration
USA: United States of America
USFDA: United States Food and Drug Administration
WD: Working days
WHO: World Health Organization
